# 793 "Minimally Invasive" Skin Grafting with Enzymatic Debridement and Autologous Skin Cell Suspension

**DOI:** 10.1093/jbcr/irac012.343

**Published:** 2022-03-23

**Authors:** Steven A Kahn, Gabriel G Gaweda, Elizabeth Halicki, Jason Hirsch, Ashley Hink, William L Hickerson, James H Holmes, Jeffrey E Carter

**Affiliations:** Medical University of South Carolina, Charleston, South Carolina; Medical University of South Carolina, Charleston, South Carolina; Medical University of South Carolina, Hanahan, South Carolina; Medical University of South Carolina, Charleston, South Carolina; Medical University of South Carolina, Charleston, South Carolina; Spectral MD, Avita Medical, AccessPro Med, Memphis, Tennessee; Atrium Health Wake Forest Baptist, Winston-Salem, North Carolina; University Medical Center- New Orleans, New Orleans, Louisiana

## Abstract

**Introduction:**

Minimally invasive surgery has become standard of care across numerous subspecialties. However, burn surgery has lagged behind; as the mainstay of treatment still involves excision with a knife and a split thickness skin graft (STSG) with a painful donor site. Enzymatic debridement with bromelain and autologous skin cell spray (ASCS) have independently been STSG use and decrease the donor site size. Due to constraints with the time course of these products only being available via studies before one was FDA approved, these technologies have not been utilized together in the United States until recently. Little literature exists regarding their use in combination. The current study characterizes a series of patients who received “minimally invasive” skin grafts with enzymatic debridement and ASCS as proof of concept.

**Methods:**

This was a retrospective study of a single academic burn center’s experience using bromelain and ASCS together. Data collection included demographics, injury characteristics, length of stay, complications, and measurements of donor sites, STSGs, and ASCS treatment. Donor site size:total area treated with ASCS and/or STSG was calculated. Length of stay (LOS) was qualitatively compared to expected using a factor of 1.1days:%TBSA, and O/E LOS ratio was calculated. Data was reported in medians with interquartile ranges. Patients with 1-30%TBSA qualified for the bromelain study and were treated according to protocol. Those deemed to have enough residual dermis were treated with ASCS, while 3^rd^ degree areas received meshed split thickness skin patch grafts with ASCS overspray.

**Results:**

Eleven patients were included in the study. Four patients received ASCS alone, while 7 patients received a meshed STSG on portions of their burn. Median burn size was 13% TBSA (IQR:5,20), while DPT+FT size was 9% TBSA (IQR:5,16). Patients had a median of 1067 sq cm (IQR:772,2183) of burn operatively treated with ASCS, and 351 sq cm (IQR:0,457) treated with meshed autograft. Donor site size (ASCS and STSG) was 225 sq cm (IQR:72,315), and ratio of donor site are to total treatment area was 0.0125 (IQR:0.01,0.32), suggesting an expansion of 80:1. Median LOS was 11 days (IQR:7,21), 0.84 days per %TBSA (IQR:0.5,1.16). Expected LOS was 14.3 days, with an O/E ratio of 0.77. Two patients developed infection; one required reoperation with STSG on half of his burned areas (5% TBSA).

**Conclusions:**

Enzymatic debridement and ASCS can be used to treat burn injury with a “minimally invasive” approach. Donor sites were much smaller than expected had they been treated with a conventional meshed STSG on deep 2^nd^ degree and 3^rd^ degree areas. The data also suggests that length of stay was lower than expected. Further study is needed to determine which subsets of patients and burn wounds are optimal for this combination of technologies.

